# Comparative LC–LTQ–MS–MS Analysis of the Leaf Extracts of *Lantana camara* and *Lantana montevidensis* Growing in Egypt with Insights into Their Antioxidant, Anti-Inflammatory, and Cytotoxic Activities

**DOI:** 10.3390/plants11131699

**Published:** 2022-06-27

**Authors:** Mariam I. Gamal El-Din, Nouran M. Fahmy, Fulin Wu, Maha M. Salem, Omar M. Khattab, Hesham R. El-Seedi, Michal Korinek, Tsong-Long Hwang, Ahmed K. Osman, Mohamed El-Shazly, Shaimaa Fayez

**Affiliations:** 1 Department of Pharmacognosy, Faculty of Pharmacy, Ain-Shams University, Cairo 11566, Egypt; mariam_gamal@pharma.asu.edu.eg (M.I.G.E.-D.); nouran_fahmy@pharma.asu.edu.eg (N.M.F.); shaimaa_fayez@pharma.asu.edu.eg (S.F.); 2 Pharmacognosy Group, Department of Pharmaceutical Biosciences, Biomedical Centre, Uppsala University, P.O. Box 591, 751 24 Uppsala, Sweden; flwu20@mails.jlu.edu.cn (F.W.); omarkhattab500@gmail.com (O.M.K.); hesham.el-seedi@farmbio.uu.se (H.R.E.-S.); 3 School of Pharmaceutical Sciences, Jilin University, Changchun 130021, China; 4 Biochemistry Division, Chemistry Department, Faculty of Science, Tanta University, Tanta 31527, Egypt; maha_salem@science.tanta.edu.eg; 5 Department of Chemistry, Faculty of Science, Menoufia University, Shebin El-Kom 32512, Egypt; 6 International Research Center for Food Nutrition and Safety, Jiangsu University, Zhenjiang 212013, China; 7 International Joint Research Laboratory of Intelligent Agriculture and Agri-Products Processing, Jiangsu Education Department, Jiangsu University, Zhenjiang 212013, China; 8 Graduate Institute of Natural Products, College of Pharmacy, Kaohsiung Medical University, Kaohsiung 80708, Taiwan; michalk@kmu.edu.tw; 9 Graduate Institute of Natural Products, College of Medicine, Chang Gung University, Kweishan, Taoyuan 33302, Taiwan; 10 Research Center for Chinese Herbal Medicine, Graduate Institute of Health Industry Technology, College of Human Ecology, Chang Gung University of Science and Technology, Kweishan, Taoyuan 33302, Taiwan; 11 Department of Anesthesiology, Chang Gung Memorial Hospital, Taoyuan 33305, Taiwan; 12 Department of Chemical Engineering, Ming Chi University of Technology, New Taipei City 243303, Taiwan; 13 Department of Botany and Microbiology, Faculty of Science, South Valley University, Qena 83523, Egypt; ahmosman2000@yahoo.com; 14 Department of Pharmaceutical Biology, Faculty of Pharmacy and Biotechnology, German University in Cairo, Cairo 11835, Egypt

**Keywords:** *Lantana camara*, *Lantana montevidensis*, antioxidant, anti-inflammatory, cytotoxicity, LC–MS–MS

## Abstract

*Lantana camara* L. and *Lantana montevidensis* Briq. (F. Verbenaceae) are invasive ornamental weeds native to the tropical regions of Africa and America. The leaves of both species have been traditionally used as infusions for treating fever, rheumatism, and cancer. LC–MS–MS-guided profiling of the methanolic extracts of the leaves of *L. camara* and *L. montevidensis* growing in Egypt led to the putative identification of 59 compounds belonging to terpenoids, flavonoids, iridoid glycosides, phenolic acids, and their derivatives. The *in-vitro* antioxidants and anti-inflammatory and anticancer activities of the two extracts were investigated. *L. camara* and *L. montevidensis* inhibited DPPH^•^ (IC_50_ = 34.01 ± 1.32 and 47.43 ± 1.74 µg/mL), ABTS^+^ (IC_50_ = 30.73 ± 1.42 and 40.37 ± 1.51 µg/mL), and superoxide anion (IC_50_ = 1.57 ± 0.19 and 1.31 ± 0.14 μg/mL) free radicals. A potent anti-inflammatory effect was observed for both species through the inhibition of elastase release in fMLF/CB-induced human neutrophils (IC_50_ = 2.40 ± 0.16 and 1.90 ± 0.07 μg/mL). The extracts showed significant cytotoxic activity against a panel of cancer cell lines with the most potent activity against Caco cells (IC_50_ = 45.65 ± 1.64 and 40.67 ± 1.52 µg/mL for *L. camara* and *L. montevidensis*, respectively). Western blotting supported by FACS analysis revealed that the extracts inhibited cancer cell proliferation, reduced metastasis, and induced apoptosis resulting in cell cycle arrest. This was achieved via increasing mRNA and protein expressions of p53 and GSK-3β as well as decreasing the expression of PI3K, Akt, and cyclin D1.

## 1. Introduction

*Lantana* sp. (Verbenaceae) is a highly invasive tropical weed that attacks more than 60% of forests worldwide [1]. The genus harbors 150 species and is native to the tropical and subtropical areas of South America, Asia, and Africa. *L. camara* L. is the most dominant species [2]. Although *Lantana* sp. is used in many countries as a decorative ornamental, the presence of pentacyclic triterpenoids, including lantadenes A and B in their leaves and seeds, has been correlated with the plant’s adverse effects, especially when ingested by animals, causing cholestasis, hepatotoxicity, and phototoxicity [2]. *Lantana* sp. is rich in phenolics, including flavonoids, iridoids, and phenylethanoids. It likewise contains alkaloids and furanonaphthoquinones. In some countries the fruits are edible and the whole *Lantana* sp. parts are used in folk medicine against fever, cancer, influenza, skin sores, chickenpox, measles, asthma, leprosy, rheumatism, and hypertension [3]. Some of these uses were proven by several studies, which reported that the leaf extracts demonstrated anti-inflammatory [4], anticancer [5], antibacterial [6], antifungal, insecticidal [7], and nematocidal activities [2]. Externally, the leaf extract is applied to the skin to heal ulcers [8] and eczema exacerbations. *L. camara* showed an ecological role by accumulating heavy metals from the soil in its roots [9]. In contrast to *L. camara* L., which is native to Africa and America, *L. montevidensis* Briq. is a small shrub indigenous to South American countries, such as Uruguay and Brazil. It is rich in phenolics, flavonoids, iridoids [10], and triterpenes. It shows antioxidant [11], antibacterial [12], and antiprotozoal [13] activities.

The accumulation of reactive oxygen species (ROS) is usually elicited by the improper balance between their production and elimination, resulting in oxidative stress, which may lead to chronic inflammation. The latter is a key factor that triggers numerous neurological [14], cardiovascular [15], hepatic [16], diabetic [17], and retinal disorders [18]. Neutrophil infiltration is one key parameter linked to inflammation and is characterized, among others, by the accumulation of superoxide anion free radicals, the release of elastases, the stimulation of serine proteases that can attack the host’s own proteins, such as lung elastin and fibronectin with the subsequent release of proinflammatory cytokines [19,20]. Excessive neutrophil activation is a profound phenomenon in the different stages of cancer development and the viral infection cycle, where the virus may act as a carcinogenic agent. Therefore, it is crucial to search for new agents from nature to inhibit neutrophil functioning, which can also indirectly suppress oxidative stress and carcinogenesis.

Cancer represents a serious public health concern with a large socioeconomic burden, especially in developing countries. By 2030, the worldwide cancer burden is expected to increase to 26 million new cases and 17 million deaths due to population expansion and aging [21]. Lung cancer has been reported to be the most prevailing diagnosed type and the leading cause of cancer death followed by breast, colorectal, and prostate cancer [22]. Surgical interventions, radiation, and chemotherapy are still the mainstay of cancer treatments despite their severe side effects arising from the indiscriminate destruction of normal cells. Therefore, there is a constant quest for the discovery of selective alternative therapies with better safety profiles, especially from nature [20,23].

AkT is a proto-oncogenic serine-threonine kinase that plays a role in cell proliferation and apoptosis. The activation of the PI3K/AkT pathway was observed in several cancer types. Therefore, the inhibition of PI3K/AkT signaling results in p53 activation, cell cycle arrest, and cancer cell apoptosis [24]. Glycogen synthase kinase 3 (GSK-3β) is a constitutively active serine/threonine kinase that is physiologically inhibited by PI3K. G1/S specific cyclin D1 is a mitogenic signal sensor whose degradation is regulated by GSK-3β and its gene expression is downregulated due to the negative regulation of GSK-3β on the oncogenic Wnt/β-catenin signaling. Cyclin D1 activity is normally intensified in cancer; therefore, cancers showing overexpression of cyclin D1 are susceptible to GSK-3β activation [25].

In continuation of our work (in finding new sources of pharmacologically active molecules from Egyptian flora [26,27]), we performed a detailed LC–MS–MS metabolome profiling of the polyphenol-rich leaf extracts of *L. camara* L. and *L. montevidensis* Briq. A comparative assessment of the extracts’ antioxidant potential, their inhibitory effects on neutrophil elastases, as well as their cytotoxic activities on cancer cell proliferation and metastasis supported by mechanistic studies on their effects on PI3K/AkT and GSK-3β/Cyclin D1 signaling pathways, were likewise investigated.

## 2. Results and Discussion

### 2.1. LC–LTQ–MS–MS Analysis and GNPS-Aided Annotation of L. camara and L. montevidensis Constituents

**Figure 1:**
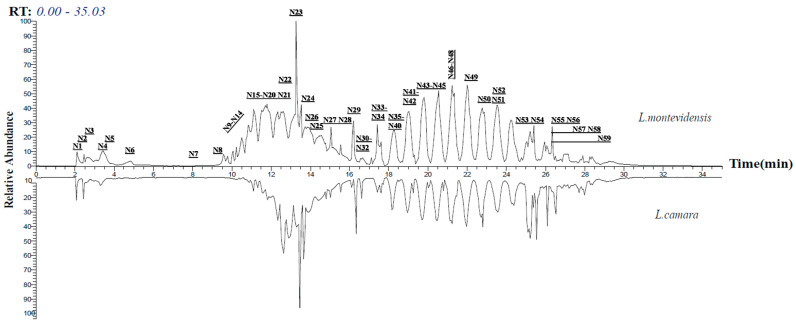
LC-MS chromatograms of *L. montevidensis* and *L. camara* in the positive ion mode prescribing the annotated metabolites from N1–N59 as exhibited in Table 1.

**Figure 2:**
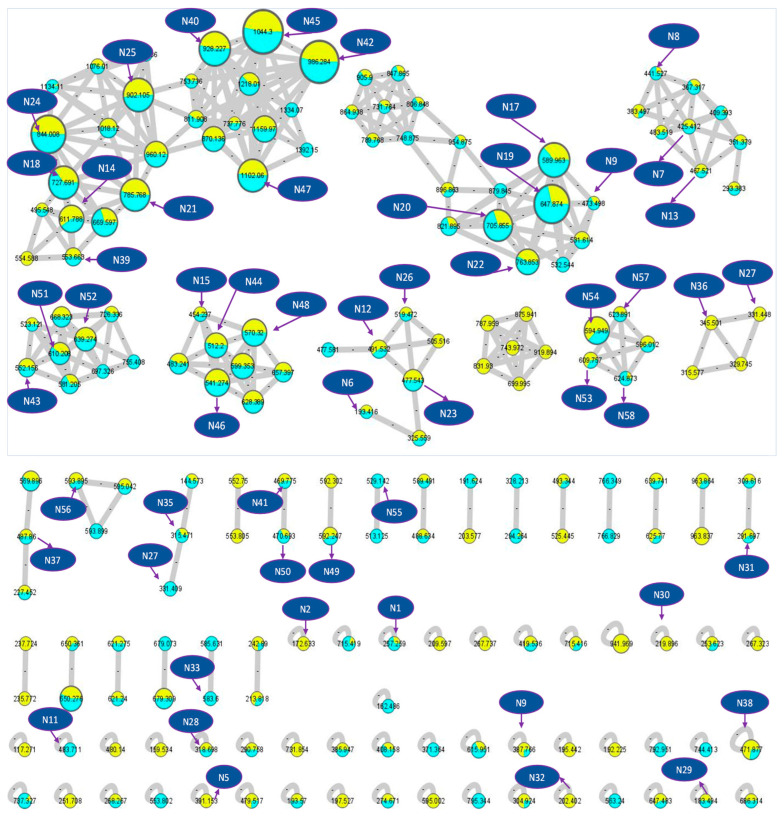
Molecular network (showing clusters of metabolites of interest) based on tandem mass spectrometry data in the positive ionization mode of *Lantana* extracts. The network is displayed as a pie chart to reflect the relative abundance of each ion in the analyzed samples. *L. camara* is indicated in yellow and *L. montevidensis* is indicated in light blue.

The metabolomic mass profiles of the two *Lantana* extracts, *L. camara* and *L. montevidensis*, were screened using the Global Natural Product Social Molecular Networking (GNPS) based on tandem mass spectrometry data (Figure 1 and Figure 2) [28,29,30] in the positive ionization mode of *Lantana* extract samples. The metabolites were represented by nodes in the molecular network, with chemically related metabolites clustered together. The network in Figure 2 was displayed as a pie chart to reflect the relative abundance of each ion in the analyzed *Lantana* extract samples. The results demonstrated a total of 157 nodes assigned for the parent ions of *L. camara* demonstrated in Figure 2 as yellow-colored nodes, of which, five parent ions matched five known standards in the GNP library (Table 1) belonging to pentacyclic triterpenes, sesquiterpenes, flavonoids, and amides. On the other side, a total of 153 nodes were assigned for the parent ions of *L. montevidensis* demonstrated in Figure 2 as blue-colored nodes, of which, four parent ions matched the known standards in the GNP library, namely palmitamide, α-humulene, coprostanone, and carminic acid. The network showed the similarities and variances of metabolites in both extracts and prescribed N1–59 as the identified metabolites (Table 1).

LC–LTQ–MS–MS analysis and molecular networking analysis resulted in the tentative identification of 59 compounds from both *Lantana* species, including 37 terpenoids, 3 iridoid glycosides, 11 flavonoids/flavonoid glycosides, 11 phenolic acids and their derivatives, among others (Table 1). The iridoid momordol and copaenol sesquiterpene were identified for the first time from the genus *Lantana*. The iridoid glycoside, durantoside, was previously identified in the roots of *L. viburnoides* [40]. Other identified compounds were previously reported from *L. camara* and *L. montevidensis*.

Terpenoids are the major metabolites produced by the genus *Lantana* [40]. Various pentacyclic triterpenoids have been reported in different *Lantana* species and are known for their wide range of pharmacological activities. Lantadenes A, D, and C, icterogenin, and pomonic acid identified in our studied *Lantana* species showed mass data following the previously reported data. Amyrin and lantabetulic acid were exclusively identified in *L. montevidensis*. 

Flavonoids represented one of the chief constituents in the genus *Lantana*, particularly flavones and flavonols [40]. Vicenin-2 (*m*/*z* 594.96) was tentatively identified in *L. camara* based on the characteristic fragmentation patterns of C-glycosides by cross-ring cleavage of the glucose moiety and the subsequent formation of the fragment ions [M+H-120]^+^ [59]. The dimethoxy flavone pectolinarigenin (*m*/*z* 315.5381) was identified in both *L. camara* and *L. montevidensis*. Flavonoid glycosides, such as pectolinarin, camaroside, and lantanoside, were detected in both species. Their identification was based on the molecular ion peaks and the respective sugars lost. A fragment ion at [M+H-162]^+^ indicated the loss of a hexoside moiety, while a fragment ion at [M+H-204]^+^ corresponded to a loss of acetylhexoside.

Simple phenolic acids, such as gallic acid, ferulic acid, and coumaric acid having *m*/*z* at 170.5321, 193.3430, and 166.5324, respectively, were detected in both *Lantana* extracts. Other phenolic acids including cistanoside C, lipedoside A, osmanthuside B, forsythoside A, calceolarioside E, isonuomioside A were also identified. Their mass fragmentation patterns were characteristic, revealing the type of the attached phenolic acid, for example, a loss of 163 Da corresponded to the loss of a caffeoyl moiety, while the loss of 147 Da corresponded to the loss of coumaric acid moiety. Iridoids such as verbascoside (*m*/*z* 624.8313, C_29_H_36_O_15_) were found in both species with fragment ions corresponding to the loss of both a caffeoyl moiety and a dehydrated rhamnose part [60].

### 2.2. Assessment of the Antioxidant Effects of L. camara and L. montevidensis Extracts

#### 2.2.1. DPPH^•^ Assay

**Figure 3:**
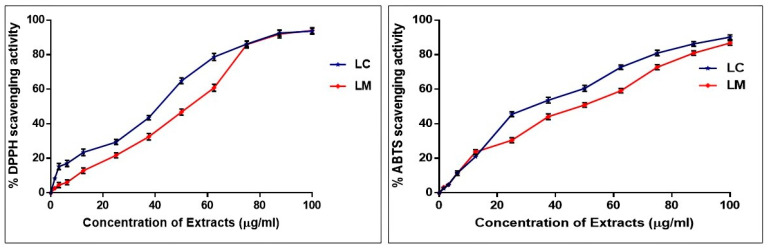
The antioxidant scavenging activity of *L. camara* and *L. montevidensis* extracts. Results were expressed as mean ± SE, (*n* = 3).

The stable DPPH^•^ radical scavenging activity assay was used to evaluate and compare the antioxidant potential of *L. camara* and *L. montevidensis* extracts. The abilities of both extracts to scavenge free radicals were assessed by measuring the change in absorbance produced by the decrease of DPPH^•^ radicals (Figure 3). The results demonstrated the dose-dependent radical scavenging capabilities of the two extracts. *L. camara* demonstrated more potent activity in scavenging DPPH^•^ free radicals with an IC_50_ value of 34.01 ± 1.32 µg/mL compared to *L. montevidensis*, which displayed an IC_50_ of 47.43 ± 1.74 µg/mL. The results were comparable to the standard L-ascorbic acid (IC_50_ 20.3 ± 1.24 µg/mL).

#### 2.2.2. ABTS^+^ Assay

The ABTS^+^ cation radical scavenging activity was measured using the decolorization assay at various concentrations of *L. camara* and *L. montevidensis*. The results showed that the ABTS^+^ cation radical scavenging activity of both extracts was concentration-dependent, similar to the DPPH assay (Figure 3). The IC_50_ scavenging capability exhibited by *L. camara* and *L. montevidensis* values were 30.73 ± 1.42 and 40.37 ± 1.51 µg/mL, respectively, compared with the standard L-ascorbic acid showing IC_50_ of 15.7 ± 1.21 µg/mL. Based on these findings, it can be concluded that both extracts exhibited a high radical scavenging capacity by reducing oxidative stress.

### 2.3. In Vitro Assessment of the Anti-Inflammatory Effects of L. camara and L. montevidensis

The methanol extracts of *L. camara* and *L. montevidensis* were investigated for their anti-inflammatory effects through the inhibition of superoxide anion generation and elastase release in fMLF/CB-induced human neutrophils. The LDH assay was likewise performed to assess the safety and/or toxicity of the tested extracts. LDH is a stable enzyme, present in all cell types, rapidly released into the cell culture medium upon the damage of the plasma membrane. The LDH assay is commonly used for the determination of cell death and cytotoxicity [61]. The results of the LDH analysis indicated the nontoxic features of both *Lantana* extracts at the tested dose of 10 μg/mL with cell viability exceeding 95% (Table 2). Therefore, both samples did not affect the growth of human neutrophils at 10 μg/mL.

*Lantana* extracts showed a dose-dependent inhibition of superoxide anion generation and elastase release in fMLF/CB-induced human neutrophils (Table 3 and Table 4). The extract of *L. montevidensis* demonstrated slightly more potent inhibition of superoxide anion with an IC_50_ of 1.31 ± 0.14 μg/mL compared to *L. camara* (IC_50_ of 1.57 ± 0.19 μg/mL). Similarly, *L. montevidensis* methanolic extract showed marked inhibition of elastase release (IC_50_ of 1.90 ± 0.07 μg/mL) compared to *L. camara* extract (IC_50_ of 2.40 ± 0.16 μg/mL) in fMLF/CB-induced human neutrophils. Both extracts at 10 μg/mL almost completely (~100%) attenuated the activation of human neutrophils, which play role in different stages of cancer and viral infections. Our results support previous studies reporting the anti-inflammatory activities of *L. camara* and *L. montevidensis* in different models [62,63]. This activity was attributed to their phytoconstituents, such as pectolinarigenin, and rutin, among others, which showed anti-inflammatory activity in previous reports [51,64,65,66].

### 2.4. In Vitro Cytotoxicity Studies on L. camara and L. montevidensis Extracts

**Figure 4:**
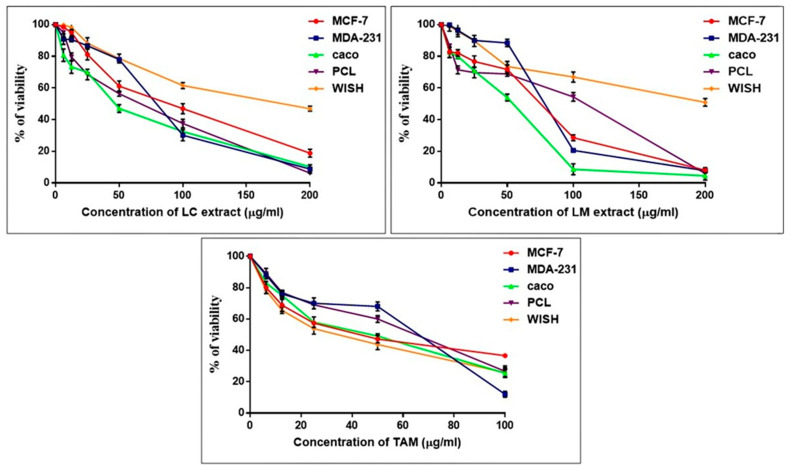
The cytotoxic effects of *L. camara*, *L. montevidensis*, and tamoxifen (standard) on different cancer cell lines. Cells were treated with various concentrations of *L*. *camara*, *L. montevidensis*, and tamoxifen for 48 h and cell viability was plotted against drugs concentration to calculate the IC_50_. Results were expressed as mean ± SE, (*n* = 5).

*L. camara* extract demonstrated cytotoxic effects when evaluated on MDA-231, Caco, PCL, and MCF-7 cancer cell lines with IC_50_ values of 74.3 ± 1.19, 45.65 ± 1.64, 52.55 ± 1.14, and 78.08 ± 1.39 µg/mL, respectively. The IC_50_ values of *L. montevidensis* extract on the same panel of cancer cell lines were 75.54 ± 1.35, 40.67 ± 1.52, 66.89 ± 1.24, and 61.43 ± 1.46 µg/mL, respectively (Figure 4). Our findings revealed the superiority of both extracts, especially on the Caco colon cancer cell line, when compared to tamoxifen (TAM—the reference drug), which had an IC_50_ value of 38.53 ± 1.25 µg/mL). The two *Lantana* extracts did not display any cytotoxicity on WISH normal cells (IC_50_ values of 166.5 ± 1.78 and 199.1 ± 1.63 µg/mL, respectively), indicating their safety on normal cells, in contrast to TAM, which showed cytotoxic effects on normal cells (IC_50_ = 30.62 ± 1.31 µg/mL) as well. Accordingly, the Caco cancer cells were selected for further molecular mechanistic studies.

#### 2.4.1. Alterations in Morphological Features of Treated Cancer Cells

**Figure 5:**
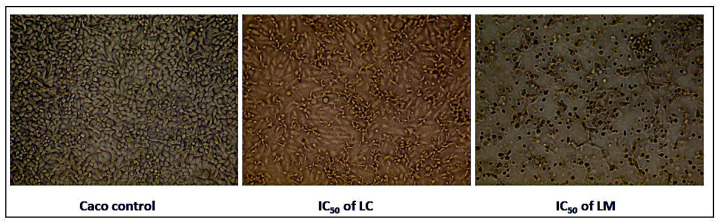
Morphological features of apoptosis in Caco cells treated with *L. camara* and *L. montevidensis* extracts (at their IC_50_ concentrations) after 48 h.

Cytotoxic agents frequently alter cell morphology, resulting in abnormal morphological changes, elevated cellular debris, and reduction in cell number. In the current study, detectable morphological features of apoptosis were observed in Caco cells treated with *L. camara* and *L. montevidensis* extracts, including cellular shrinkage, reduction in cell number, detachment of the cells, cell rounding, and condensation of the cytoplasm. However, the morphology of the untreated cells appeared normal and confluent (Figure 5).

#### 2.4.2. Analysis of the Cell Cycle

**Figure 6:**
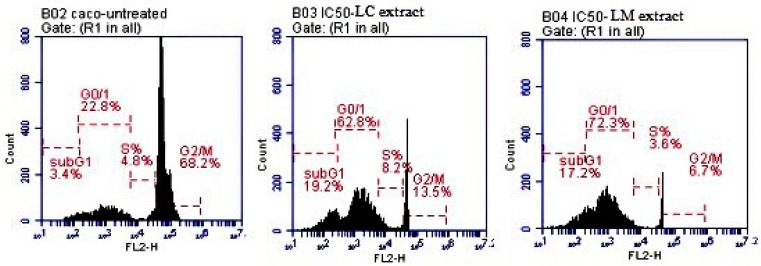
Cell cycle phases of Caco cells treated with *L. camara* and *L. montevidensis* extracts (at their IC_50_ concentrations) after 48 h of treatment.

Cell cycle arrest occurs when the PI3K/Akt protein kinases are inhibited. The activation of GSK-3β and the blockade of the cyclin D1 signaling pathway reduces the proliferation and metastasis of the Caco cancer cell line. When compared with the untreated cells, both extracts increased the percentage of cells in the sub-G_0_/G_1_ phase (the phase at which the cells wait before entering the cell cycle to duplicate) in Caco cells at the IC_50_ level. When the number of cells in this phase rises, the cell cycle stops, and division as well as DNA replication are impossible. Figure 6 showed that at the IC_50_ concentrations, the two *Lantana* extracts caused cell cycle arrest at rates of 19.2% and 17.2% in the sub-G_0_/G_1_ phase, respectively, compared to the untreated Caco cells (3.4%). These findings showed that *Lantana* extracts could inhibit the PI3K/AkT and GSK-3β/cyclin D1 signaling pathways, as well as trigger apoptosis by arresting the cell cycle in the sub-G_0_/G_1_ phase [67,68].

#### 2.4.3. qRT-PCR Assessment

**Figure 7:**
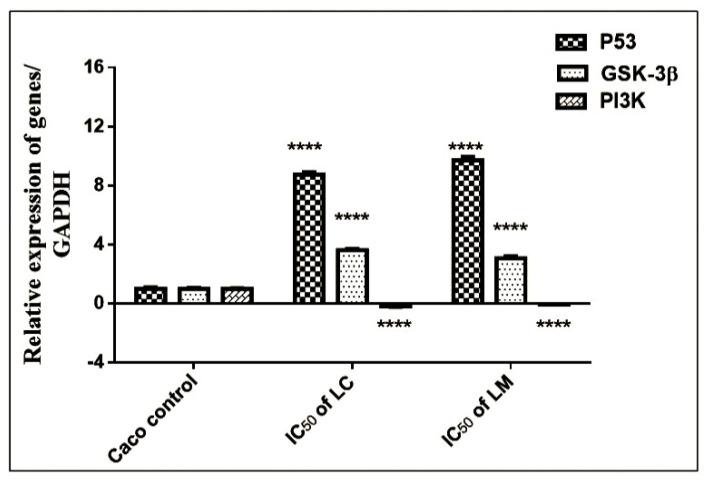
Relative expression of p53, GSK-3β, and PI3K in Caco cells. Results were expressed as mean ± SE, (*n* = 3). **** *p* < 0.0001 is considered significant compared to the Caco control untreated cells.

The mRNA expressions of the p53 (apoptotic markers), PI3K, and GSK-3β (proliferative markers) genes in the Caco cell line were measured using qRT-PCR. The expressions of p53 and GSK-3β were significantly (*p* < 0.0001) enhanced in cells treated with both *Lantana* extracts compared to the untreated cells. Similarly, PI3K gene expression was downregulated in *Lantana*-treated cells as compared with the untreated cells (Figure 7). Therefore, *Lantana* extracts had the potential to limit cancer cell proliferation and metastasis causing cell cycle arrest and apoptosis, which was clarified by the overexpression of p53 and GSK-3β as well as the downregulation of the PI3K gene [69].

#### 2.4.4. Immunoblotting Assay

**Figure 8:**
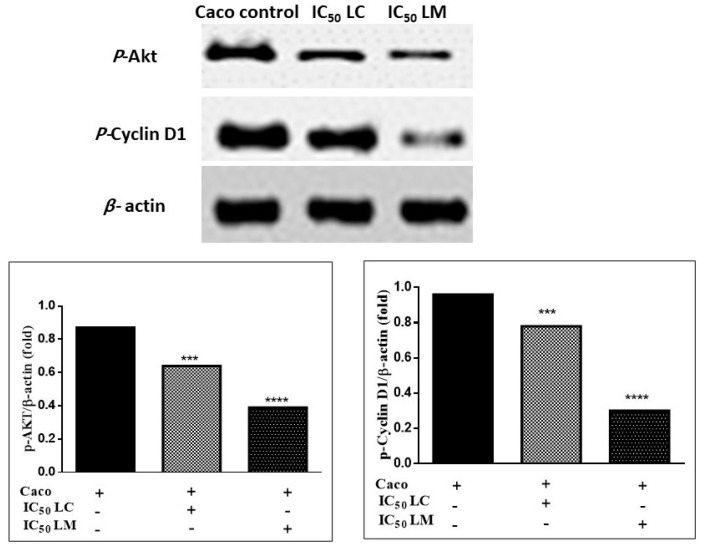
Western blot analysis of *L. camara* and *L. montevidensis* extracts in Caco cells. Results were expressed as mean ± SE, (*n* = 3). *** *p* < 0.001 and **** *p* < 0.0001 is considered significant compared to the Caco control untreated cells. Bands were relatively expressed to β-actin protein (internal control) (Figures S7 and S8) by western blot analysis.

Compared to the untreated cells, both *Lantana* extracts resulted in a significant decrease in Akt protein kinase (Figures S1–S3) and cyclin D1 (Figures S4–S6) in Caco cells (Figure 8). These findings revealed that the extracts suppressed PI3K resulting in Akt inhibition via dephosphorylation. The dephosphorylation of Akt activates p53, which subsequently arrests the cell cycle. It likewise stimulated GSK-3β, which inhibited cyclin D1, leading to reduced cellular proliferation, angiogenesis, and metastasis.

## 3. Materials and Methods

### 3.1. Plant Collection and Extraction

**Figure 9:**
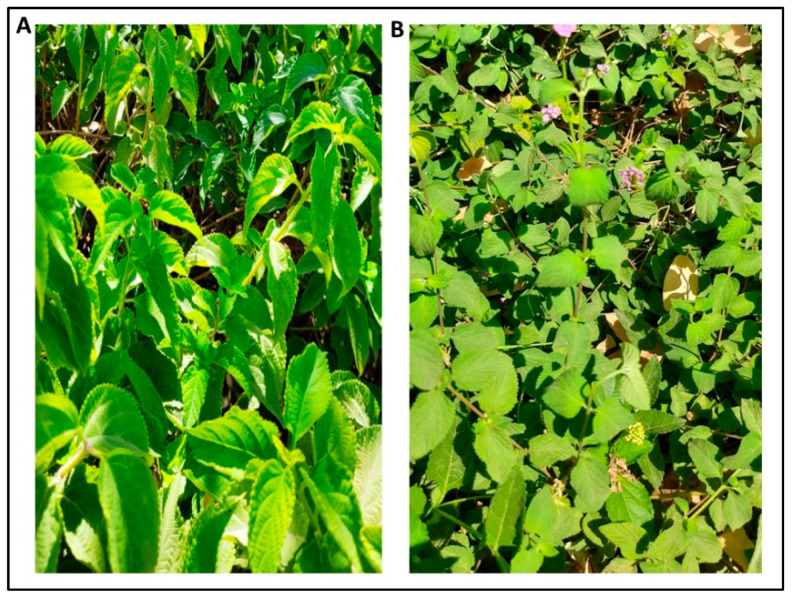
Photos of the collected fresh leaves of *L. camara* (**A**) and *L. montevidensis* (**B**).

Fresh young leaves of *L. camara* L. (Syn. *Camara vulgaris* Benth.) and *L. montevidensis* (Spreng.) Briq. (Verbenaceae) were collected from South Valley University Garden and Aswan Botanical Garden, Aswan, Egypt, respectively, in March 2020. The woody shrubs of *L. camara* L. were grown in sandy soil, irrigated by groundwater wells, and supplied by natural fertilizers. Nevertheless, *L. montevidensis* woody shrubs were grown in loam soil, irrigated by river Nile fresh water, and supplied by natural fertilizers. Photos of the collected fresh leaves are demonstrated in Figure 9. Authentication was achieved by Therese Labib, Consultant at El-Orman Botanic Garden, Giza, Egypt. Voucher specimens were deposited at the herbarium at the Department of Pharmacognosy, Faculty of Pharmacy, Ain Shams University, Egypt (PHG-V-LC-244), and (PHG-V-LM-245). The dried plant materials (500 g each) were extracted by successive maceration (3X-2 L each) in methanol (Al-Brouj, Giza, Egypt). The macerate was filtered, evaporated, and concentrated in vacuo at 45 °C to yield 2.17 g and 2.34 g of dark brown extracts of *L. camara* and *L. montevidensis*, respectively. The methanol extracts were subsequently defatted using *n*-hexane (Al-brouj, Giza, Egypt), evaporated, freeze-dried, then stored in amber-colored bottles for further chemical analysis and biological investigations.

### 3.2. LC–LTQ–MS–MS Analysis of L. camara and L. montevidensis Extracts

The defatted methanol extract was analyzed using LC–MS–MS. A Shimadzu LC-10 HPLC with a Grace Vydac Everest Narrowbore C-18 column (100 mm × 2.1 mm i.d., 5 µm, 300 Å). LC–MS, connected to an LTQ Linear Ion Trap MS (Thermo Finnigan, San Jose, CA) was utilized with a mass range of 100–2000 m/z. A 2 µL sample was injected using an autosampler. A 35 min method was used as follows: 5 min isocratic run using 5% acetonitrile (Acn) and 0.05% formic acid (FA), then a gradient was run for 25 min until 95% AcN 0.05% FA. Finally, there was 5 min of conditioning the column with 5% AcN and 0.05% FA. The data were processed and analyzed using foundation 3.1_Xcalibur_3.1.6610. Furthermore, the raw data files were converted to mzXML format using MSConvert from the ProteoWizard suite [70]. The molecular network was created using the Global Natural Products Social Molecular Networking (GNPS) online workflow [28,29]. The spectra in the network were then searched against the GNPS spectral libraries and published data.

### 3.3. In Vitro Assessment of the Antioxidant Activities of L. camara and L. montevidensis Extracts

#### 3.3.1. DPPH^•^ Free Radical Scavenging

The DPPH^•^ assay was used to examine the free radical scavenging capacity of the two extracts according to the method published by Burits and Bucar [71] with certain modifications. Briefly, various concentrations of LC and LM (1.56–100 µg/mL) were added and mixed gently with 975 µL of (0.003 g%) DPPH^•^ in methanol. The reaction mixture absorbance (A) was measured at 515 nm using a Jenway 6305 UV/Vis spectrophotometer after 1 h of dark incubation at room temperature. A reaction without the extract was carried out as a control. As a positive control, L-ascorbic acid (20–100 µg/mL) was utilized, and the DPPH^•^ radical scavenging activity (%) was measured using the equation:

$$\mathrm{DPPH}\;\mathrm{radical}\;\mathrm{scavenging}\;\mathrm{activity}\;\%=\frac{A\;\mathrm{control}-A\;\mathrm{sample}}{A\;\mathrm{control}}\times 100$$

#### 3.3.2. ABTS^+^ Radical Scavenging Activity

The ABTS cation radical (ABTS^+^) scavenging activity of the two extracts was measured according to Re et al. [72]. The ABTS solution (14 mM) reacted with the potassium persulfate solution (4.9 mM) for 16 h in the dark. ABTS^+^ cation radicals were produced. The ABTS^+^ solution was diluted with distilled water to achieve an absorbance of 0.734 at 734 nm before use. Then, 975 µL of ABTS^+^ solution was added to 25 µL of the two *Lantana* extracts containing different concentrations (from 1.56 to 100 µg/mL). Absorbance was measured at 734 nm after 4 min of dark incubation and compared to the control. Ascorbic acid (20–100 µg/mL) was employed as a positive control and the ABTS^+^ cation radical scavenging activity (%) was estimated using the following equation:

$$\mathrm{ABTS}\;\mathrm{radical}\;\mathrm{scavenging}\;\mathrm{activity}\;\left(\%\right)=\frac{A\;\mathrm{control}-A\;\mathrm{sample}}{A\;\mathrm{control}}\times 100$$

### 3.4. In Vitro Assessment of the Anti-Inflammatory Activity of L. camara and L. montevidensis

#### 3.4.1. Preparation of Human Neutrophils

Blood was collected from healthy human donors (20–35 years old) using a protocol conducted according to the guidelines of the Declaration of Helsinki, and approved by the institutional review board at Chang Gung Memorial Hospital (IRB no. 201902217A3). Informed consent was obtained from all subjects involved in the study. Neutrophils were isolated as previously described [73]. In brief, the blood samples were processed by dextran sedimentation and Ficoll–Hypaque centrifugation, followed by hypotonic lysis of contaminating red blood cells [74]. The segregated neutrophils were suspended and stored in pH 7.4 Ca^2+^-free Hank’s balanced salt solution (HBSS) at 4 °C before the experiments. Next, the Wright–Giemsa stain was applied to confirm the purity of the suspension of neutrophils. Finally, the cellular viability of >98% was confirmed by the trypan blue exclusion method.

#### 3.4.2. Lactate Dehydrogenase (LDH) Assay

The damage or toxicity to the cells may be expressed as LDH release because the cell membrane loses its integrity, and LDH stored in the cytoplasm is released outside the cell. The cell viability assay based on LDH release was performed to ensure the safety of the extracts on human neutrophils [75]. In brief, human neutrophils (6 × 10^5^ cells/mL) were preheated at 37 °C for 5 min in 1 mM CaCl_2_ and were incubated with the tested extracts for 15 min. Total LDH released from the cells was incubated with 0.1% of Triton X-100 for 30 min to completely cause cell lysis. Cells were centrifuged at 4 °C for 200× *g* for 8 min, an LDH reagent was added to the supernatant, and the mixture was incubated in the dark at room temperature for 30 min. The absorbance was then measured at 492 nm, and the LDH release was calculated and compared to the total LDH release set as 0%; untreated cells were set as 100%.

#### 3.4.3. Measurement of Superoxide Generation

Ferricytochrome *c* was used to evaluate the superoxide release in human neutrophils [76]. The method was described in a previous study [77]. Human neutrophils (6 × 10^5^ cells/mL) were incubated in HBSS containing ferricytochrome *c* (0.6 mg/mL) and CaCl_2_ (1 mM). The mixture was equilibrated at 37 °C for 5 min and then was incubated with the tested samples or DMSO (control) for 5 min. Cells were primed by cytochalasin B (CB, 1 μg/mL) and were activated with formyl-methionyl-leucyl-phenylalanine (fMLF, 100 nM) for 10 min. The absorbance was constantly monitored at 550 nm using a double-beam, six-cell positioned spectrophotometer Hitachi U-3010 with constant stirring (Hitachi Inc., Tokyo, Japan). Calculations were based on the differences in absorbance in the presence or absence of superoxide dismutase (SOD, 100 U/mL) divided by the extinction coefficient for ferricytochrome *c* reduced form (ε = 21.1/mM/10 mm). LY294002 was used as the positive control.

#### 3.4.4. Measurement of Elastase Release

Elastase release was measured by degranulation of azurophilic granules in human neutrophils [78]. Human neutrophils (6 × 10^5^ cells/mL) were equilibrated with elastase substrate MeO-Suc-Ala-Ala-Pro-Val-*p*-nitroanilide (100 μM) in HBSS supplemented with CaCl_2_ (1 mM) at 37 °C for 2 min and then were incubated with samples or DMSO (control) for 5 min. Human neutrophils were activated by 100 nM fMLF and 0.5 μg/mL CB and the changes in the absorbance at 405 nm were continuously monitored by a spectrometer (Hitachi U-3010, Tokyo, Japan) to record the elastase release. The results were expressed as the percent of elastase release in the fMLF/CB-activated drug-free control system. LY294002 was used as the positive control.

### 3.5. In Vitro Cytotoxicity Investigation of L. camara and L. montevidensis Extracts

#### 3.5.1. Cell Lines Maintenance and Treatment

The triple-negative breast cancer cell line (MDA-231), pancreatic cancer cell line (PCL), estrogen receptor-positive breast cancer cell line (MCF-7), colon cancer cell line (Caco), and WISH normal cell line were seeded with (1 × 10^4^ cells/well) separately using complete media containing Dulbecco’s Modified Eagle supplemented with 10% heat-inactivated fetal bovine serum (FBS, GIBCO, Langley, OK, USA; cat. no. 10099133), 1% penicillin/streptomycin (Thermo Fisher Scientific, Waltham, MA, USA; cat. no. SV30082) in 5% CO_2_ incubator and 95% humidified environment at 37 °C. All cell lines were provided by the Centre of Excellence for Research in Regenerative Medicine and its Applications, Alexandria University, Egypt. Cell lines were incubated with *Lantana* extracts at several concentrations (0–200 µg/mL) and tamoxifen (TAM) as a standard chemotherapeutic drug (0–100 µg/mL) for 48 h. The viability of cells was determined using the tetrazolium 3-(4,5-dimethylthiazol-2-yl)-2,5-diphenyl-tetrazolium bromide (MTT) assay (Gibco-BRL, New York, NY, USA) [79].

#### 3.5.2. Cell Morphology Study

Briefly, 1 × 10^5^ of the Caco cell line was seeded in a 6-well plate, incubated for 24 h, then treated with *Lantana* extracts at their IC_50_ concentrations. After 48 h of incubation, morphological alterations of the treated and untreated cells were evaluated and captured using an inverted light microscope (Olympus, Tokyo, Japan).

#### 3.5.3. Cell Cycle Examination

Flow cytometry was used to analyze cell cycle phases using an Accuri C6 flow cytometer (Becton Dickinson BD, Franklin Lakes, NJ, USA) on Caco cells 1 × 10^5^ that were trypsinized, centrifuged at 5000 rpm at 4 °C, washed with cold phosphate buffer saline (PBS), and fixed with cold absolute ethanol, as described by Noser et al. and Darzynkiewicz et al. [80,81].

#### 3.5.4. Quantitative Real-Time PCR (qRT-PCR)

The Caco 1 × 10^5^ control and treated cells were trypsinized, centrifuged at 4500 rpm at 4 °C, and washed with PBS. The pelleted cells were subjected to RNA extraction and transcription to cDNA as described by Kvastad et al. [82]. The expressions of p53, GSK-3β, and PI3K mRNA were measured using Applied qPCR Biosystems (Foster City, CA, USA) on treated and control cells according to Livak and Schmittgen [83]. The primer sequences were designed using primer 3 plus as shown in Table 5.

#### 3.5.5. Western Blot Analysis

The method of Mruk and Cheng was used for immunoblotting assay [84]. Proteins were separated from Caco control and treated cells using cold RIPA lysis buffer and were quantified using Bradford [85]. Equal amounts of proteins (20 mg) were separated and transferred to a polyvinylidene difluoride (PVDF) membrane. After blocking the membrane, the primary antibodies, phospho-AkT (ab81283) and cyclin D1 (ab134175), were added and incubated with it. Then, the primary antibodies were removed, carefully washed several times, and incubated with the secondary antibody horseradish peroxidase (HRP) (ab205718). The bands were visualized using enhanced chemiluminescence (ECL) detection kit (Promega, Madison, WI, USA). A gel documentation system (Geldoc-it, UVP, Cambridge, UK), was applied for data analysis using TotalLab analysis software, Newcastle upon Tyne, England, www.totallab.com, (Ver.1.0.1).

### 3.6. Statistical Analysis

Results are expressed as mean ± SEM value of at least three independent measurements unless otherwise specified. The 50% inhibitory concentration (IC_50_) was calculated from the dose-response curve obtained by plotting the percentage of inhibition versus concentrations (linear function, Microsoft Office, Redmond, WA, U.S). Statistical analysis was performed by Student’s *t*-test (Sigma Plot, Systat software, Systat Software Inc., San Jose, CA, USA, anti-inflammatory assay). Values with * *p* < 0.05, ** *p* < 0.01, *** *p* < 0.001 were considered statistically significant.

## 4. Conclusions

LC–MS–MS-guided metabolic profiling of *L. camara* and *L. montevidensis* extracts resulted in the tentative identification of 59 compounds belonging to different phytochemical classes including pentacyclic triterpenes, flavonoids, and phenolic acids. In vitro studies revealed that *Lantana* species displayed potent radical scavenging and anti-inflammatory activities through the inhibition of elastase release in fMLF/CB-induced human neutrophils. The extracts, despite their encouraging safety profile on normal human cells, exhibited potent cytotoxic effects on a wide array of cancer cell lines, especially against Caco cells. *Lantana* extracts induced apoptosis and triggered cell cycle arrest. They inhibited the proliferation and metastasis of cancer cells by downregulating the PI3K/AkT signaling cascade and reducing cyclin D1 levels via the activation of GSK-3β. Our findings imply that *L. camara* and *L. montevidensis* crude extracts could be valuable sources for further research as potential anticancer agents.

## Figures and Tables

**Table 1:** LC–LTQ–MS–MS dereplication of the alcoholic leaf extracts of *L. camara* (*Lc*) and *L. montevidensis* (*Lm*) crude extracts.

No.	Compound Name	R_t_	Formula	*m*/*z*	MS^2^	Relative Abundance		Chemical Class	Ref.
						*Lc*	*Lm*		
1.	Palmitamide	1.69	C_16_H_33_NO	257.2312	239.1328, 201.1358, 187.1268, 103.0043, 88.9936	11.84	4.58	FA amide	GNPS
2.	Gallic acid	2.67	C_7_H_6_O_5_	171.5321	152.9517, 85.9147	17.85	12.44	Phenolic acid	[31]
3.	α-Humulene	2.76	C_15_H_24_	202.3955	155.9594, 141.9675, 127, 9391	9.65	4.14	Monocyclic sesquiterpene	GNPS
4.	*p*-Coumaric acid	3.73	C_9_H_8_O_3_	166.5324	148.9633, 119.9389	5.95	3.97	Phenolic acid	[32]
5.	Theveside	3.88	C_16_H_22_O_11_	391.1981	373.0371, 355.0253, 279.0401, 228.9559, 210.9715, 192.9794, 148.935	7.15	6.09	Iridoid	[33]
6.	Ferulic acid	4.82	C_10_H_10_O_4_	193.3430	174.9286, 162.9635, 116.9951	15.32	14.58	Phenolic acid	[34]
7.	Lamiridoside	8.01	C_17_H_26_O_12_	425.3458	276.1333, 218.0855, 160.0576	12.52	12.14	Iridoid	[35]
8.	Momorodol	9.84	C_26_H_49_O_5_	441.5767	292.1333, 234.0912, 176.1231, 172.0572, 160.0817	9.09	6.65	Triterpene	[36]
9.	Pomolic acid	10.01	C_30_H_48_O_4_	473.4977	455.1928, 397.1318, 321.1171, 227.0642, 169.0692	11.08	3.49	Triterpene	[13,37]
10.	Coprostanone	10.12	C_27_H_46_O	387.3893	369.0617, 355.1737, 351.2124, 313.1629, 269.1862	6.31	3.55	Triterpene	GNPS
11.	Dihydrixyolean-enoic acid (Hederagenin)	10.12	C_30_H_49_O_4_	473.5281	455.1928, 397.1318, 379.2358, 339.2030, 321.1171, 245.1248, 227.0642, 203.1588	11.08	8.26	Triterpene	[37,38]
12.	Carminic acid	10.18	C_22_H_20_O_13_	491.3225	315.1465, 300.1916, 159.1022	32.62	21.71	Flavonoid	GNPS
13.	Amyrin	10.42	C_30_H_50_O	467.4973	334.1802, 276.1421, 218.0966, 160.013	-	6.39	Triterpene	[38]
14.	Rutin	10.77	C_27_H_30_O_16_	611.6093	553.2927, 477.2678, 317.1439, 301.124, 271.1095	45.86	39.98	Flavonoid	[31]
15.	Lantadene C	11.32	C_35_H_54_O_5_	553.61	535.2781, 525.3694, 495.2407, 477.2509, 401.1971, 301.1317	21.51	24.82	Triterpene	[39]
16.	Calceolarioside E	11.88	C_23_H_26_O_11_	479.4901	461.0790, 443.0788, 425.1241, 317.0097, 299.1252, 263.1182, 162.982	17.17	11.54	Phenolic acid	[37]
17.	Triterpene glycoside derivative	12	-	589.7351	513.2347, 455.2542, 437.2231, 379.2318, 285.1443	57.74	73.83	Triterpene glycoside	[39,40]
18.	Triterpene glycoside derivative	12.4	-	727.4208	709.4453, 669.3836, 670.4085, 651.4137, 611.3975, 593.3708	56.84	73.05	Triterpene glycoside	
19.	Triterpene glycoside derivative	12.5	-	647.7673	571.2557, 513.2347, 455.2542, 437.2231, 379.2318, 285.1443	73.45	100	Triterpene glycoside	[41]
20.	Triterpene glycoside derivative	12.8	-	705.8645	571.2820, 513.2588, 629.2966, 437.2466, 495.2869, 455.2817, 285.1443	55.65	75.45	Triterpene glycoside	[39,40]
21.	Triterpene glycoside derivative	13.4	-	785.4552	727.4422, 709.4453, 669.3836, 670.4085, 651.4137, 611.3975, 593.3708,	66.14	67.99	Triterpene glycoside	
22.	Durantoside	13.13	C_35_H_40_O_19_	763.9281	687.3165, 629.3169, 571.0399, 513.3507, 437.2202, 285.1336	39.41	52.87	Iridoid	[40]
23.	Dihydroxy-dimethoxyflavone-O-glucopyranoside (Camaroside)	13.45	C_23_H_24_O_11_	477.4684	459.1729, 357.1318, 315.2007, 301.1745	47.17	36.08	Flavonoid	[37]
24.	Triterpene glycoside derivative	14.27	-	843.9122	785.4807, 767.5064, 727.5031, 709.4918	54.35	54.97	Triterpene glycoside	
25.	Triterpene glycoside derivative	14.83	-	901.9307	543.5972, 825.6067, 767.5857, 709.5566	39.59	38.59	Triterpene glycoside	
26.	Lantanoside	14.68	C_25_H_26_O_12_	519.6086	459.1729, 357.1318, 315.2007, 301.1745,	10.89	10.75	Flavonoid	[42]
27.	Cirsiliol/ Trihydroxy-dimethoxyflavone	15.05	C_17_H_14_O_7_	331.4301	316.1003, 285.1243, 271.1618, 151.052	14.30	17.36	Flavonoid	[43,44]
28.	Hexahydroxyflavone (Gossypetin)	15.07	C_15_H_10_O_8_	318.6483	283.10, 242.90, 183.05, 169, 156.90, 109, 96.92	6.03	4.86	Flavonoid	[37]
29.	Caffeic acid	16.26	C_9_H_9_O_4_	181.5156	162.9605, 135.0327, 107.0433, 59.0440	23.17	16.72	Phenolic acid	[44]
30.	Copaenol	16.44	C_15_H_25_O	221.7963	203.0473, 175.0733, 161.0492	13.81	5.39	Sesquiterpene	[45]
31.	Catechin	16.81	C_15_H_15_O_6_	291.6731	273.0806, 255.1193, 217.0495, 147.0402	12.79	9.06	Flavonoid	[46]
32.	Methyl-hydroxylantanolate	16.84	C_31_H_49_O	500.7488	482.2634, 469.2448, 401.2497, 317.1457	5.21	3.48	Triterpeme	[47]
33.	Ursangilic acid	17.06	C_36_H_54_O_6_	583.6300	565.2075, 485.2668, 467.3068, 449.361	6.46	8.04	Triterpene	[40,48]
34.	Benzalkonium chloride	17.65	C_21_H_38_N^+^	304.8513	212.1338, 90.9176	3.6	-	Ammonium Compound	GNPS
35.	Dihydroxy-dimethoxyflavone (Pectolinarigenin)	18.05	C_17_H_14_O_6_	315.5381	300.0404, 282.0015, 269.0966, 121.0294	26.17	22.87	Flavonoid	[37,49]
36.	Dihydroxy-trimethoxyflavone	18.20	C_18_H_17_O_7_	345.4969	330.1257, 313.1737, 285.16, 151.0042	17.83	4.92	Flavonoid	[50]
37.	Lantaninilic acid/Lantoic acid	18.50	C_30_H_46_O_5_	487.6703	469.2092, 451.2662, 433.2741, 405.2914, 259.1011	18.75	10.55	Triterpene	[37,51]
38.	Camarin	18.62	C_30_H_46_O_4_	471.865	451.2739, 433.3931, 423.2796, 405.3678, 395.294, 313.2488, 271.192	46.93	15.27	Triterpene	[52,53]
39.	Stigmasterol acetate	18.77	C_31_H_50_O_2_	454.2024	328.1359, 299.1358. 270.1251, 241.083, 211.9450, 182.9712	16.83	18.26	Triterpene	[35]
40.	Triterpene glycoside derivative	18.99	-	927.9218	851.6035, 793.5519, 735.5273, 677.4890, 635.4446	28.27	34.97	Triterpene	
41.	Pomonic acid	19.24	C_30_H_46_O_4_	469.6669	451.2739, 395.294, 313.2488	20.68	17.92	Triterpene	[37]
42.	Triterpene glycoside derivative	19.80	-	985.9156	909.6604, 851.6151, 793.5680, 735.5282, 677.4925	35.37	43.41	Triterpene	
43.	Lantadene A	20.02	C_35_H_52_O_5_	552.7491	524.3465, 506.4606, 478.4638, 316.2659	26.88	31.96	Triterpene	[54]
44.	Lantanone	20.14	C_32_H_48_O_5_	512.3184	482.4986, 425.2136, 357.3943, 328.3304, 299.2942, 270.1839	5.81	47.77	Triterpene	[37]
45.	Lablaboside derivative	20.51	-	1043.9023	941.7347, 793.5754, 647.5658, 473.4372, 389.2796, 331.2671	37.08	45.57	Triterpene glycoside	
46.	Lantadene D	21.21	C_34_H_52_O_5_	541.2581	511.6045, 425.2182, 357.3688, 328.3118, 299.2821, 270.2123, 241.122	6.53	56.70	Triterpene	[39]
47.	Lablaboside A	21.26	C_54_H_87_O_23_	1102.8948	941.7347, 793.5754, 647.5658, 473.4372, 389.2796, 331.2671	37.39	47.76	Triterpene glycosides	[41]
48.	Icterogenin/Lantacin	21.64	C_35_H_52_O_6_	570.2877	551.2625, 451.2778, 405.2828, 357.3562, 299.2313, 241.1121	59.43	67.93	Triterpene	[39,40,52]
49.	Osmanthuside B	22.33	C_29_H_36_O_13_	592.267	574.2937, 524.5654, 447.2928, 389.3079, 331.2425, 273.1571	30.85	32.81	Phenolic acid	[55]
50.	Hydroxyoleanonic acid/Lantabetulic acid	22.78	C_30_H_48_O_4_	470.0257	452.2639, 434.261, 396.2762, 307.0825	-	17.92	Triterpene	[37,42,56]
51.	Isonuomioside A	23.01	C_28_H_34_O_15_	610.2413	591.4889, 531.5561, 447.2902, 389.2357, 339. 1718, 243.0484	49.29	48.15	Phenolic acid	[42]
52.	Cistanoside C	23.31	C_30_H_38_O_15_	639.2013	621.2913, 552.4019, 505.355, 447.2967	44.41	44.59	Phenolic acid	[55]
53.	Lipedoside A	25.42	C_29_H_36_O_14_	609.6940	591.3207, 559.3793, 531.3948, 515.344	49.29	48.15	Phenolic acid	[55]
54.	Apigenin-6,8-di-C-glycoside (Vicenin 2)	25.48	C_27_H_30_O_15_	594.9509	576.4872, 534.3077, 474.3816, 642.376, 317.2311, 236.2413	100	54.74	Flavonoid	[55]
55.	Camarinic acid	26.57	C_35_H_62_O_3_	529.1427	283.2026, 256.3454, 246.3058, 242.3309, 163.1626, 149.1549	15.61	6.3	Triterpene	[57]
56.	Pheophorbide A	26.59	C_35_H_36_N_4_O_5_	593.9152	533.391, 473.4104, 461.4372, 433.4519	30.61	54.74	Chlorophyll derivative	[35]
57.	Pectolinarigenin-*O*-rutinoside (Pectolinarin)	27.88	C_29_H_34_O_15_	623.886	605.3220, 545.3717, 459.3893, 395.3564, 367.3008	14.18	9.35	Flavonoid	[37]
58.	Verbascoside/Forsythoside A	27.89	C_29_H_36_O_15_	624.8313	606.2446, 546.3597, 397.3595, 284.3636, 266.3377	9.16	6.87	Phenolic acid	[52,58]
59.	Vanillic acid	31.12	C_12_H_6_O_4_	169.8434	150.9395, 140.9568, 123.0144, 108.9833	16.99	12.36	Phenolic acid	[44]

**Table 2:** The effects of *Lantana* extracts on the release of lactate dehydrogenase (LDH) in human neutrophils.

Extract	Cell Viability (%)
*L. camara*	95.88 ± 4.60
*L. montevidensis*	97.11 ± 2.89

Percentage of cell viability (%) at 10 μg/mL. Results are presented as mean ± SEM (*n* = 3).

**Table 3:** Effects of *Lantana* extracts on superoxide anion generation in fMLF/CB-induced human neutrophils.

Extract	IC_50_^a^	Inhibition% (1 μg/mL)	Inhibition% (3 μg/mL)	Inhibition% (10 μg/mL)
*L. camara*	1.57 ± 0.19 μg/mL	24.80 ± 4.53 **	91.94 ± 4. 90 ***	100.49 ± 1.14 ***
*L. montevidensis*	1.31 ± 0.14 μg/mL	29.90 ± 4.28 ***	97.70 ± 0.26 ***	100.92 ± 0.29 ***
LY294002 ^b^	2.41 ± 0.26 μM			

Results are presented as mean ± SEM (*n* = 3–5). ** *p* < 0.01, *** *p* < 0.001 compared to the control (DMSO). ^a^ Concentration necessary for 50% inhibition (IC_50_). ^b^ LY294002, the PI3K inhibitor, was used as a positive control with potent suppressive effects.

**Table 4:** Effects of *Lantana* extracts on elastase release in fMLF/CB-induced human neutrophils.

Extract	IC_50_^a^	Inhibition% (1 μg/mL)	Inhibition% (3 μg/mL)	Inhibition% (10 μg/mL)
*L. camara*	2.40 ± 0.16 μg/mL	9.95 ± 2.32 *	64.22 ± 6.33 ***	109.24 ± 5.15 ***
*L. montevidensis*	1.90 ± 0.07 μg/mL	16.68 ± 0.04 ***	80.82 ± 4.68 ***	115.84 ± 2.02 ***
LY294002 ^b^	3.18 ± 0.57 μM			

Results are presented as mean ± SEM (*n* = 3–5). * *p* < 0.05, *** *p* < 0.001 compared to the control (DMSO). ^a^ Concentration necessary for 50% inhibition (IC_50_). ^b^ LY294002, the PI3K inhibitor, was used as the positive control with potent suppressive effects.

**Table 5:** Primer sequences used for qRT-PCR.

Gene	Forward Primer (/5–/3)	Reverse Primer (/5–/3)
p53	TAACAGTTCCTGCATGGGCGGC	AGGACAGGCACAAACACGCACC
GSK-3β	CCGACTAACACCACTGGAAGCT	AGGATGGTAGCCAGAGGTGGAT
PI3K	GCTCTCTCACTGCATACATTGT	AGTCACAGCTGTATTGGTCG
GAPDH	TGTGTCCGTCGTGGATCTGA	CCTGCTTCACCACCTTCTTGA

## Data Availability

Data are available in the manuscript and Supplementary Material.

## Supplementary Materials

The following supporting information can be downloaded at: https://www.mdpi.com/article/10.3390/plants11131699/s1, Figure S1. β- actin expression level for samples; Figure S2. Computerized analysis of β-actin protein expression level for samples; Figure S3. Anti P-AkT antibody protein expression level for samples; Figure S4. Computerized analysis of anti P-AkT antibody protein expression level for samples; Figure S5. Dendograms of anti- P-AkT antibody protein expression level for samples 1 (A), 2 (B), and 3 (C); Figure S6. Anti-cyclin D1 antibody protein expression level for samples; Figure S7. Computerized analysis of anti-cyclin D1 antibody protein expression level for samples; Figure S8. Dendograms of anti-cyclin D1 antibody protein expression level for samples 1 (A), 2 (B), and 3 (C).

## References

[1] Sharma G.P., Raghubanshi A.S., Singh J.S. (2005). *Lantana* invasion: An overview. Weed Biol. Manag..

[2] Negi G.C.S., Sharma S., Vishvakarma S.C.R., Samant S.S., Maikhuri R.K., Prasad R.C., Palni L.M.S. (2019). Ecology and Use of *Lantana camara* in India. Bot. Rev..

[3] Saxena M., Saxena J., Khare S. (2012). A brief review on: Therapeutical values of *Lantana camara* plant. Int. J. Pharm. Life Sci..

[4] dos Santos Lencina J., Bonfa Moslaves I.S., de Araujo Isaias Muller J., Carvalho R., Amianti C., Bonfim I., Alves F.M., Carollo C.A., Candeloro L., dos Santos Júnior A.A. (2021). *Lantana canescens* (Kunth) inhibits inflammatory and hyperalgesic responses in murine models. J. Ethnopharmacol..

[5] Dawood A.S., Chua L.S., Tan T.S., Alshemary A.F. (2021). Apoptotic mechanism of lantadene A from *Lantana camara* leaves against prostatic cancer cells. Egypt. J. Chem..

[6] Amany R., Yara K., Tharwat R., Gamal H., Khaulood H. (2021). Multidrug-resistant *Staphylococcus* bacteria isolated from pregnant women and the antimicrobial effect of *Lantana camara* L. different extracts. Egypt. J. Exp. Biol..

[7] Qureshi H., Anwar T., Ali Q., Haider M.Z., Habib N., Fatima S., Waseem M., Bibi Y., Arshad M., Adkins S.W. (2021). Isolation of natural herbicidal compound from *Lantana camara*. Int. J. Environ. Anal. Chem..

[8] Edem G.D., Okon K.A., Essien S.I., Bassey E.-O.I. (2021). *Lantana camara*: A potent influential factor in improving the gastric mucosa of wistar rats ravaged by ulcer. Biol. Clin. Sci. Res. J..

[9] Ibrahim M.A., Sabti M.Z., Mousa S.H. (2021). In vitro accumulation potentials of heavy metals in big-sage (*Lantana camara* L.) plant. DYSONA-Life Sci..

[10] Mohamed N.M., Makboul M.A., Farag S.F., Tarawneh A.H., Khan S.I., Brooks T.A., Wang Y.-H., Ross S.A. (2017). Iridoid and phenylpropanoid glycosides from the roots of *Lantana montevidensis*. Med. Chem. Res..

[11] Sousa E.O., Rocha J.B.T., Barros L.M., Barros A.R.C., Costa J.G.M. (2013). Phytochemical characterization and in vitro antioxidant properties of *Lantana camara* L. and *Lantana montevidensis* Briq. Ind. Crops Prod..

[12] Barreto F.S., Sousa E.O., Rodrigues F.F.G., Costa J.G.M., Campos A.R. (2010). Antibacterial Activity of *Lantana camara* Linn *Lantana montevidensis* Brig Extracts from Cariri-Ceara, Brazil. J. Young Pharm..

[13] Mohamed N.M., Makboul M.A., Farag S.F., Jain S., Jacob M.R., Tekwani B.L., Ross S.A. (2016). Triterpenes from the roots of *Lantana montevidensis* with antiprotozoal activity. Phytochem. Lett..

[14] Hald A., Lotharius J. (2005). Oxidative stress and inflammation in Parkinson’s disease: Is there a causal link?. Exp. Neurol..

[15] García N., Zazueta C., Aguilera-Aguirre L. (2017). Oxidative Stress and Inflammation in Cardiovascular Disease. Oxidative Med. Cell. Longev..

[16] Li S., Hong M., Tan H.-Y., Wang N., Feng Y. (2016). Insights into the Role and Interdependence of Oxidative Stress and Inflammation in Liver Diseases. Oxidative Med. Cell. Longev..

[17] Burgos-Morón E., Abad-Jiménez Z., Martínez de Marañón A., Iannantuoni F., Escribano-López I., López-Domènech S., Salom C., Jover A., Mora V., Roldan I. (2019). Relationship between Oxidative Stress, ER Stress, and Inflammation in Type 2 Diabetes: The Battle Continues. J. Clin. Med..

[18] Rivera J.C., Dabouz R., Noueihed B., Omri S., Tahiri H., Chemtob S. (2017). Ischemic Retinopathies: Oxidative Stress and Inflammation. Oxidative Med. Cell. Longev..

[19] Kawabata K., Hagio T., Matsuoka S. (2002). The role of neutrophil elastase in acute lung injury. Eur. J. Pharmacol..

[20] Fayez S., Ayoub I.M., Mostafa N.M., Moussa A.Y., Gamal El-Din M.I., El-Shazly M., Chakraborti S. (2021). Nutraceuticals in Cancer Therapy. Handbook of Oxidative Stress in Cancer: Therapeutic Aspects.

[21] Thun M.J., DeLancey J.O., Center M.M., Jemal A., Ward E.M. (2010). The global burden of cancer: Priorities for prevention. Carcinogenesis.

[22] von Meyenfeldt M. (2005). Cancer-associated malnutrition: An introduction. Eur. J. Oncol. Nurs..

[23] Wang C.-D., Yuan C.-F., Bu Y.-Q., Wu X.-M., Wan J.-Y., Zhang L., Hu N., Liu X.-J., Zu Y., Liu G.-L. (2014). Fangchinoline inhibits cell proliferation via Akt/GSK-3beta/cyclin D1 signaling and induces apoptosis in MDA-MB-231 breast cancer cells. Asian Pac. J. Cancer Prev..

[24] Luo J., Manning B.D., Cantley L.C. (2003). Targeting the PI3K-Akt pathway in human cancer: Rationale and promise. Cancer Cell.

[25] Takahashi-Yanaga F., Sasaguri T. (2008). GSK-3β regulates cyclin D1 expression: A new target for chemotherapy. Cell. Signal..

[26] El-Seedi H.R., Burman R., Mansour A., Turki Z., Boulos L., Gullbo J., Goransson U. (2013). The traditional medical uses and cytotoxic activities of sixty-one Egyptian plants: Discovery of an active cardiac glycoside from *Urginea maritima*. J. Ethnopharmacol..

[27] El-Seedi H.R., Yosri N., Khalifa S.A.M., Guo Z., Musharraf S.G., Xiao J., Saeed A., Du M., Khatib A., Abdel-Daim M.M. (2021). Exploring natural products-based cancer therapeutics derived from egyptian flora. J. Ethnopharmacol..

[28] El-Garawani I.M., El-Sabbagh S.M., Abbas N.H., Ahmed H.S., Eissa O.A., Abo-Atya D.M., Khalifa S.A.M., El-Seedi H.R. (2020). A newly isolated strain of *Halomonas* sp.(HA1) exerts anticancer potential via induction of apoptosis and G2/M arrest in hepatocellular carcinoma (HepG2) cell line. Sci. Rep..

[29] El-Garawani I., Hassab El-Nabi S., El Kattan A., Sallam A., Elballat S., Abou-Ghanima S., El Azab I.H., El-Seedi H.R., Am Khalifa S., El-Shamy S. (2021). The ameliorative role of *Acacia senegal* gum against the oxidative stress and genotoxicity induced by the radiographic contrast medium (ioxitalamate) in albino rats. Antioxidants.

[30] Elrasoul A.S.A., Mousa A.A., Orabi S.H., Mohamed M.A.E.-G., Gad-Allah S.M., Almeer R., Abdel-Daim M.M., Khalifa S.A.M., El-Seedi H.R., Eldaim M.A.A. (2020). Antioxidant, anti-inflammatory, and anti-apoptotic effects of *Azolla pinnata* ethanolic extract against lead-induced hepatotoxicity in rats. Antioxidants.

[31] Sousa E.O., Miranda C.M., Nobre C.B., Boligon A.A., Athayde M.L., Costa J.G. (2015). Phytochemical analysis and antioxidant activities of *Lantana camara* and *Lantana montevidensis* extracts. Ind. Crops Prod..

[32] Sharma O.P., Sharma S., Pattabhi V., Mahato S.B., Sharma P.D. (2007). A review of the hepatotoxic plant *Lantana camara*. Crit. Rev. Toxicol..

[33] Cittadini M.C., García-Estévez I., Escribano-Bailón M.T., Rivas-Gonzalo J.C., Valentich M.A., Repossi G., Soria E.A. (2018). Modulation of fatty acids and interleukin-6 in glioma cells by South American tea extracts and their phenolic compounds. Nutr. Cancer.

[34] Singh M., Tamma R.V., Nigg H.N. (1989). HPLC identification of allelopathic compounds from *Lantana camara*. J. Chem. Ecol..

[35] Van Wyk B.-E. (2017). A review of African medicinal and aromatic plants. Med. Aromat. Plants World-Afr..

[36] Begum S., Ahmed M., Siddiqui B.S., Khan A., Saify Z.S., Arif M. (1997). Triterpenes, a sterol and a monocyclic alcohol from *Momordica charantia*. Phytochemistry.

[37] Lata R.R. (2020). Extraction and Bioactivity of Organic Extracts of *Lantana camara* Leaves. Master’s Thesis.

[38] Singh S.K., Tripathi V.J., Singh R.H. (1990). 3β, 24-Dihydroxyolean-12-en-28-oic acid, a pentacyclic triterpene acid from *Lantana indica*. Phytochemistry.

[39] Shamsee Z.R., Al-Saffar A.Z., Al-Shanon A.F., Al-Obaidi J.R. (2019). Cytotoxic and cell cycle arrest induction of pentacyclic triterpenoides separated from *Lantana camara* leaves against MCF-7 cell line in vitro. Mol. Biol. Rep..

[40] Sousa E.O., Costa J.G. (2012). Genus *Lantana*: Chemical aspects and biological activities. Rev. Bras. Farmacogn..

[41] Matsuda H., Li Y., Murakami T., Yamahara J., Yoshikawa M. (1998). Protective effects of oleanolic acid oligoglycosides on ethanol- or indomethacin-induced gastric mucosal lesions in rats. Life Sci..

[42] de Sousa E.O., de Almeida S.C., Damasceno S.S., Nobre C.B., da Costa J.G.M. (2018). *Lantana camara* L. and *Lantana montevidensis* (Spreng.) Briq. Medicinal and Aromatic Plants of South America.

[43] Nagao T., Abe F., Kinjo J., Okabe H. (2002). Antiproliferative constituents in plants 10. Flavones from the leaves of *Lantana montevidensis* B RIQ. and consideration of structure–activity relationship. Biol. Pharm. Bull..

[44] Darwish R.S., El-Banna A.A., Ghareeb D.A., El-Hosseny M.F., Seadawy M.G., Dawood H.M. (2022). Chemical profiling and unraveling of anti-COVID-19 biomarkers of red sage (*Lantana camara* L.) cultivars using UPLC-MS/MS coupled to chemometric analysis, in vitro study and molecular docking. J. Ethnopharmacol..

[45] Weyerstahl P., Wahlburg H.C., Marschall H., Rustaiyan A. (1993). Terpenes and terpene derivatives, XXXII. New cadinene and bisabolene derivatives from the essential oil of *Pulicaria gnaphalodes*. Liebigs Ann. Chem..

[46] Rahma N.A., Rohman A. (2022). UPLC MS/MS Profile and Antioxidant Activities from Nonpolar Fraction of Patiwala (*Lantana camara*) Leaves Extract. Separations.

[47] Hart N., Lamberton J., Sioumis A., Suares H. (1976). New triterpenes of *Lantana camara*. A comparative study of the constituents of several taxa. Aust. J. Chem..

[48] Hussain H., Hussain J., Al-Harrasi A., Shinwari Z.K. (2011). Chemistry of some species genus *Lantana*. Pak. J. Bot..

[49] Makboul M.A., Attia A.A., Farag S.F., Mohamed N.M., Ross S.A. (2014). Chemical constituents with free-radical scavenging activity from the leaves of *Lantana montevidensis* (Spreng.) Briq. Phcog J..

[50] Gaillard P., HautevIlle M., Picq M., Duclos M.-C., Dubois M., Prigent A.-F. (1996). Selective inhibition of rat heart cAMP phosphodiesterases by lipophilic C-methyl-2-phenyl-4H-1-benzopyran-4-ones (C-methylflavones). Chem. Pharm. Bull..

[51] Begum S., Ayub A., Shaheen Siddiqui B., Fayyaz S., Kazi F. (2015). Nematicidal triterpenoids from *Lantana camara*. Chem. Biodivers..

[52] Begum S., Zehra S.Q., Siddiqui B.S., Fayyaz S., Ramzan M. (2008). Pentacyclic triterpenoids from the aerial parts of *Lantana camara* and their nematicidal activity. Chem. Biodivers..

[53] Begum S., Zehra S.Q., Wahab A., Siddiqui B.S. (2006). Triterpenoidal secondary metabolites from *Lantana camara* Linn. Helv. Chim. Acta.

[54] Begum S., Ayub A., Qamar Zehra S., Shaheen Siddiqui B., Iqbal Choudhary M. (2014). Leishmanicidal triterpenes from *Lantana camara*. Chem. Biodivers..

[55] Abdel-Hady H., El-Sayed M.M., Abdel-Hady A.A., Hashash M.M., Abdel-Hady A.M., Aboushousha T., Abdel-Hameed E.-S.S., Abdel-Lateef E.E.-S., Morsi E.A. (2018). Nephroprotective Activity of methanolic extract of *Lantana camara* and squash (*Cucurbita pepo*) on cisplatin-induced nephrotoxicity in rats and identification of certain chemical constituents of Lantana camara by HPLC-ESI-MS. Pharmacogn. J..

[56] Sharma O.P., Singh A., Sharma S. (2000). Levels of lantadenes, bioactive pentacyclic triterpenoids, in young and mature leaves of *Lantana camara* var. aculeata. Fitoterapia.

[57] Siddiqui B.S., Raza S.M., Begum S., Siddiqui S., Firdous S. (1995). Pentacyclic triterpenoids from *Lantana camara*. Phytochemistry.

[58] Wollenweber E., Dorr M., Muniappan R., Siems K. (1997). Flavonoid aglycones and triterpenoids from the leaf exudate of *Lantana camara* and *Lantana montevidensis*. Biochem. Syst. Ecol..

[59] Sánchez-Rabaneda F., Jáuregui O., Casals I., Andrés-Lacueva C., Izquierdo-Pulido M., Lamuela-Raventós R.M. (2003). Liquid chromatographic/electrospray ionization tandem mass spectrometric study of the phenolic composition of cocoa (*Theobroma cacao*). J. Mass Spectrom..

[60] Cardinali A., Pati S., Minervini F., D’Antuono I., Linsalata V., Lattanzio V. (2012). Verbascoside, isoverbascoside, and their derivatives recovered from olive mill wastewater as possible food antioxidants. J. Agric. Food Chem..

[61] Kumar P., Nagarajan A., Uchil P.D. (2018). Analysis of cell viability by the lactate dehydrogenase assay. Cold Spring Harb. Protoc..

[62] Wu P., Song Z., Wang X., Li Y., Li Y., Cui J., Tuerhong M., Jin D.-Q., Abudukeremu M., Lee D. (2020). Bioactive triterpenoids from *Lantana camara* showing anti-inflammatory activities in vitro and in vivo. Bioorg. Chem..

[63] Silva T., Suffredini I., Ricci E., Fernandes S., Gonçalves V., Romoff P., Lago J., Bernardi M. (2015). Antinociceptive and anti-inflammatory effects of *Lantana camara* L. extract in mice. Rev. Bras. Plantas Med..

[64] Ghosh S., Das Sarma M., Patra A., Hazra B. (2010). Anti-inflammatory and anticancer compounds isolated from *Ventilago madraspatana* Gaertn., *Rubia cordifolia* Linn. and *Lantana camara* Linn. J. Pharm. Pharmacol..

[65] Yuting C., Rongliang Z., Zhongjian J., Yong J. (1990). Flavonoids as superoxide scavengers and antioxidants. Free Radic. Biol. Med..

[66] Xu G.-H., Kim Y.-H., Choo S.-J., Ryoo I.-J., Yoo J.-K., Ahn J.-S., Yoo I.-D. (2009). Chemical constituents from the leaves of *Ilex paraguariensis* inhibit human neutrophil elastase. Arch. Pharmacal Res..

[67] Deng S., Dai G., Chen S., Nie Z., Zhou J., Fang H., Peng H.J.B. (2019). Dexamethasone induces osteoblast apoptosis through ROS-PI3K/AKT/GSK3β signaling pathway. Biomed. Pharmacother..

[68] Yang K., Guo Y., Stacey W.C., Harwalkar J., Fretthold J., Hitomi M., Stacey D.W. (2006). Glycogen synthase kinase 3 has a limited role in cell cycle regulation of cyclin D1 levels. BMC Cell Biol..

[69] Gao X., Li X., Ho C.-T., Lin X., Zhang Y., Li B., Chen Z. (2020). Cocoa tea (*Camellia ptilophylla*) induces mitochondria-dependent apoptosis in HCT116 cells via ROS generation and PI3K/Akt signaling pathway. Food Res. Int..

[70] MS-Convert. http://proteowizard.sourceforge.net/download.html.

[71] Burits M., Bucar F. (2000). Antioxidant activity of *Nigella sativa* essential oil. Phytother. Res..

[72] Re R., Pellegrini N., Proteggente A., Pannala A., Yang M., Rice-Evans C. (1999). Antioxidant activity applying an improved ABTS radical cation decolorization assay. Free Radic. Biol. Med..

[73] Yang S.-C., Chung P.-J., Ho C.-M., Kuo C.-Y., Hung M.-F., Huang Y.-T., Chang W.-Y., Chang Y.-W., Chan K.-H., Hwang T.-L. (2013). Propofol inhibits superoxide production, elastase release, and chemotaxis in formyl peptide–activated human neutrophils by blocking formyl peptide receptor 1. J. Immunol..

[74] Bøyum A., Løvhaug D., Tresland L., Nordlie E. (1991). Separation of leucocytes: Improved cell purity by fine adjustments of gradient medium density and osmolality. Scand. J. Immunol..

[75] Korinek M., Hsieh P.-S., Chen Y.-L., Hsieh P.-W., Chang S.-H., Wu Y.-H., Hwang T.-L. (2021). Randialic acid B and tomentosolic acid block formyl peptide receptor 1 in human neutrophils and attenuate psoriasis-like inflammation in vivo. Biochem. Pharmacol..

[76] Babior B.M., Kipnes R.S., Curnutte J.T. (1973). Biological defense mechanisms. The production by leukocytes of superoxide, a potential bactericidal agent. J. Clin. Investig..

[77] Chen C.-Y., Liaw C.-C., Chen Y.-H., Chang W.-Y., Chung P.-J., Hwang T.-L. (2014). A novel immunomodulatory effect of ugonin U in human neutrophils via stimulation of phospholipase C. Free Radic. Biol. Med..

[78] Hwang T.-L., Leu Y.-L., Kao S.-H., Tang M.-C., Chang H.-L. (2006). Viscolin, a new chalcone from *Viscum coloratum*, inhibits human neutrophil superoxide anion and elastase release via a cAMP-dependent pathway. Free Radic. Biol. Med..

[79] Dash S.K., Ghosh T., Roy S., Chattopadhyay S., Das D. (2014). Zinc sulfide nanoparticles selectively induce cytotoxic and genotoxic effects on leukemic cells: Involvement of reactive oxygen species and tumor necrosis factor alpha. J. Appl. Toxicol..

[80] Darzynkiewicz Z., Halicka H.D., Zhao H. (2010). Analysis of cellular DNA content by flow and laser scanning cytometry. Polyploidization Cancer.

[81] Noser A.A., Abdelmonsef A.H., El-Naggar M., Salem M.M. (2021). New Amino Acid Schiff Bases as Anticancer Agents via Potential Mitochondrial Complex I-Associated Hexokinase Inhibition and Targeting AMP-Protein Kinases/mTOR Signaling Pathway. Molecules.

[82] Kvastad L., Werne Solnestam B., Johansson E., Nygren A., Laddach N., Sahlén P., Vickovic S., Bendigtsen S.C., Aaserud M., Floer L. (2015). Single cell analysis of cancer cells using an improved RT-MLPA method has potential for cancer diagnosis and monitoring. Sci. Rep..

[83] Livak K.J., Schmittgen T.D. (2001). Analysis of relative gene expression data using real-time quantitative PCR and the 2−ΔΔCT method. Methods.

[84] Mruk D.D., Cheng C.Y. (2011). Enhanced chemiluminescence (ECL) for routine immunoblotting: An inexpensive alternative to commercially available kits. Spermatogenesis.

[85] Bradford M.M. (1976). A rapid and sensitive method for the quantitation of microgram quantities of protein utilizing the principle of protein-dye binding. Anal. Biochem..

