# Supplementary material for: Comparative LC–LTQ–MS–MS Analysis of the Leaf Extracts of Lantana camara and Lantana montevidensis Growing in Egypt with Insights into Their Antioxidant, Anti-Inflammatory, and Cytotoxic Activities
Source: Plants (Basel). 2022 Jun 27;11(13):1699. doi: 10.3390/plants11131699 (PMC9269492; doi:10.3390/plants11131699)
Supplement: Supplementary file 1 [file plants-11-01699-s001.zip › plants-1770724-supplementary.pdf]

Supplementary information

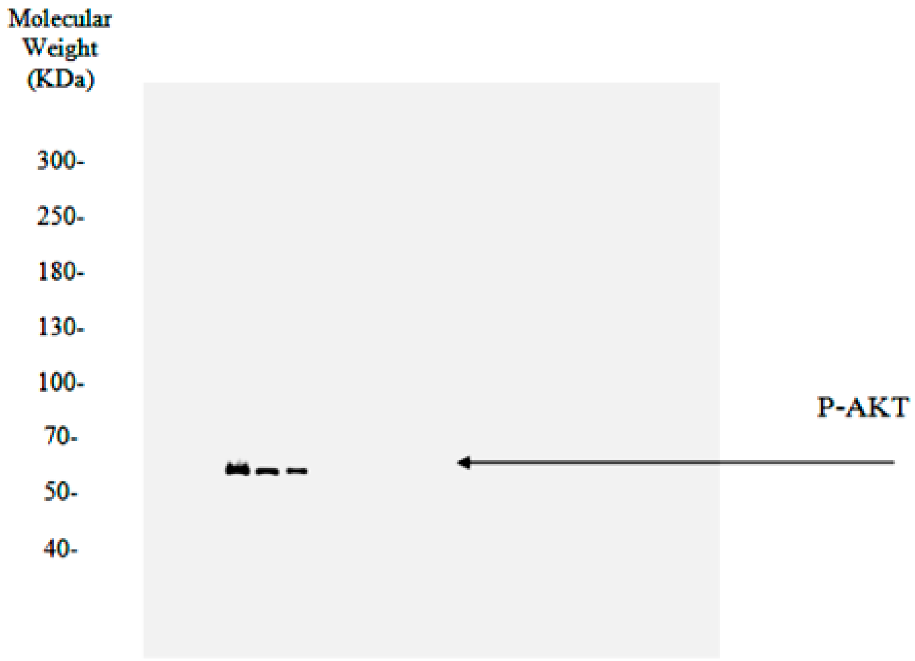

Figure S1. Anti P-AKT antibody protein expression level for samples.

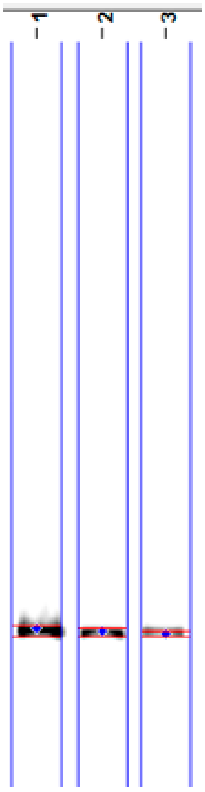

Figure S2. Computerized analysis of anti P-AKT antibody protein expression level for samples.

A

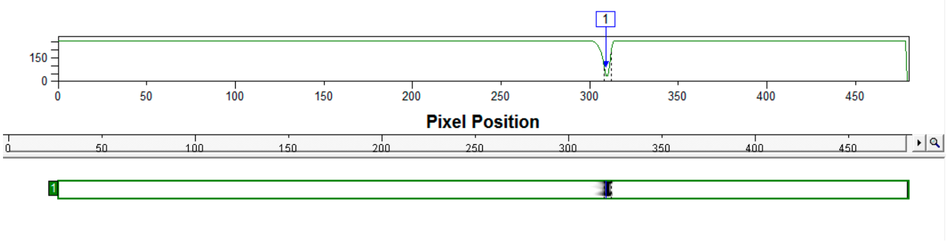

B

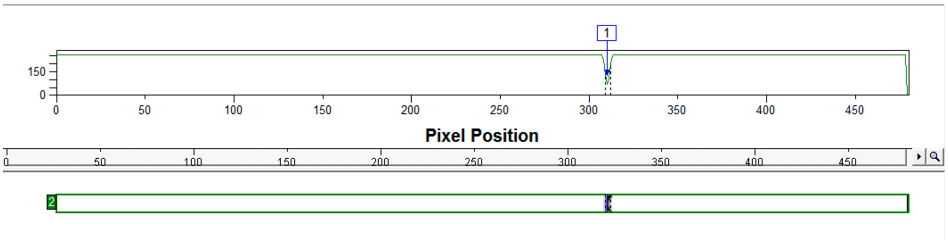

C

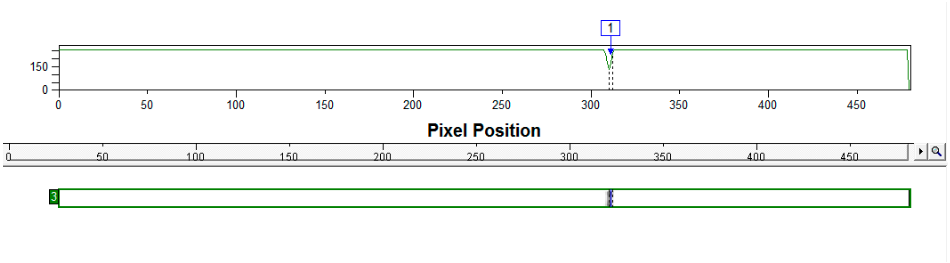

Figure S3. Dendograms of anti- P-AKT antibody protein expression level for samples 1 (A), 2 (B), and 3 (C).

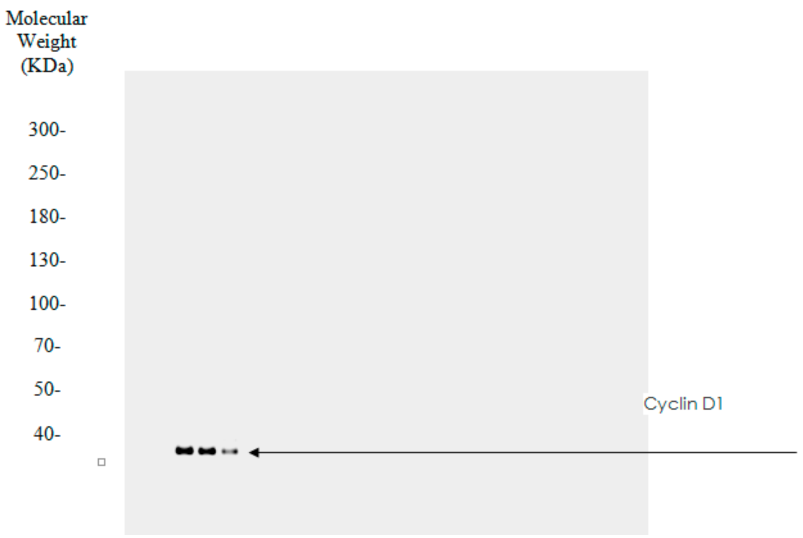

Figure S4. Anti-cyclin D1 antibody protein expression level for samples.

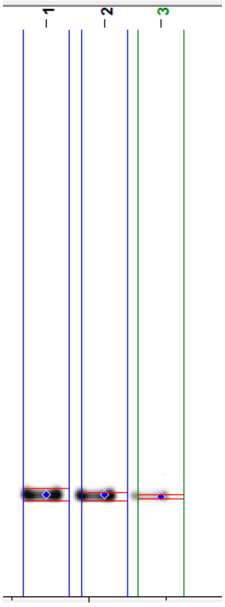

Figure S5. Computerized analysis of anti-cyclin D1 antibody protein expression level for samples.

A

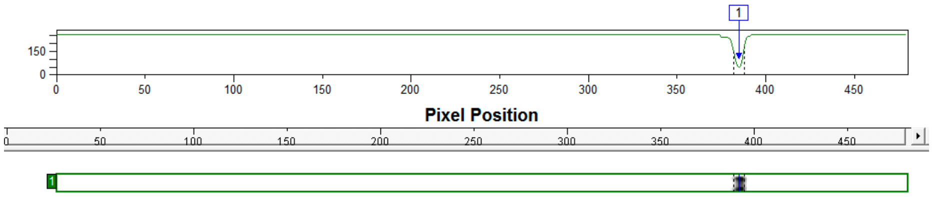

B

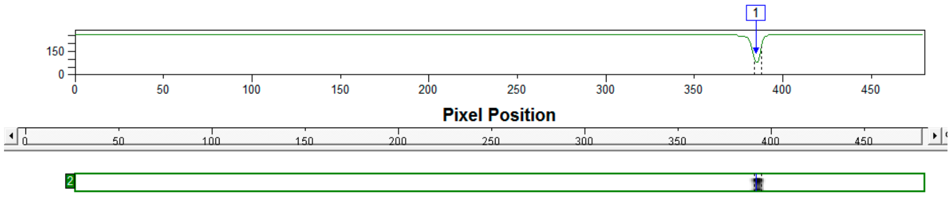

C

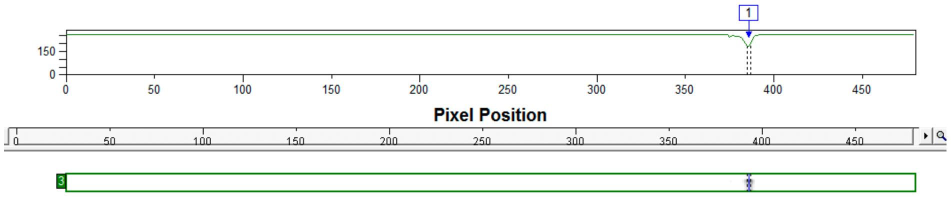

Figure S6. Dendograms of anti-cyclin D1 antibody protein expression level for samples 1 (A), 2 (B), and 3 (C).

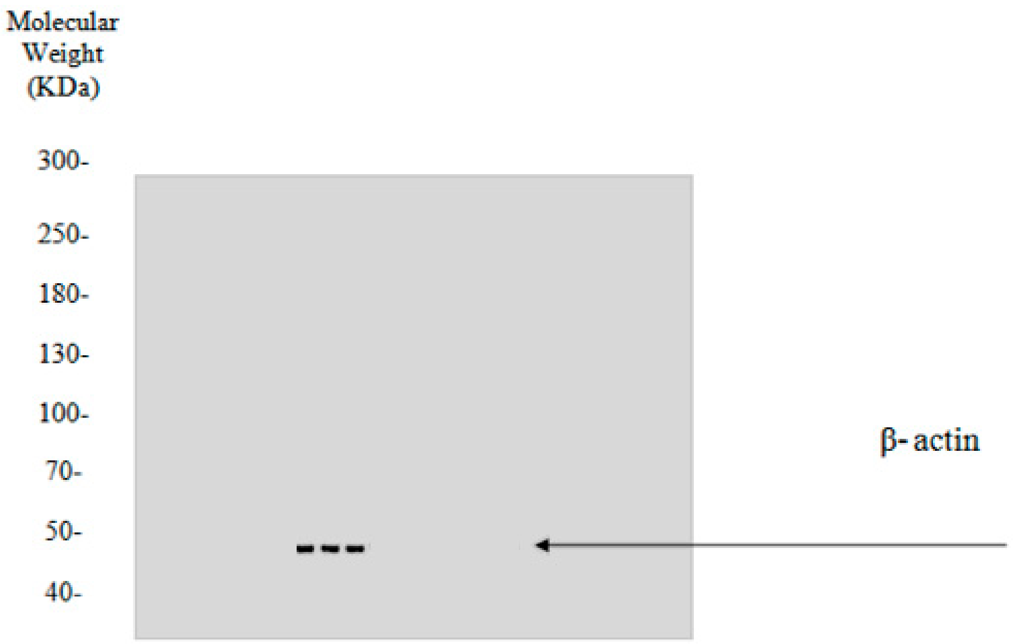

Figure S7. β- actin expression level for samples

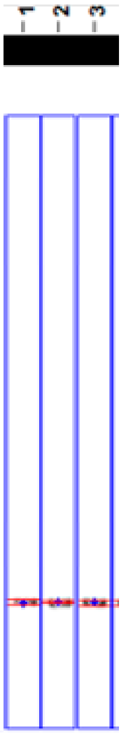

Figure S8. Computerized analysis of β-actin protein expression level for samples.
